# The bacterial cell division protein fragment ^E^FtsN binds to and activates the major peptidoglycan synthase PBP1b

**DOI:** 10.1074/jbc.RA120.015951

**Published:** 2021-01-13

**Authors:** Adrien Boes, Frederic Kerff, Raphael Herman, Thierry Touze, Eefjan Breukink, Mohammed Terrak

**Affiliations:** 1 InBioS-Centre d'Ingénierie des Protéines, Liège University, Liège, Belgium; 2 Université Paris-Saclay, CEA, CNRS, Institute for Integrative Biology of the Cell (I2BC), Gif-sur-Yvette, France; 3 Membrane Biochemistry and Biophysics, Department of Chemistry, Faculty of Science, Utrecht University, Utrecht, The Netherlands

**Keywords:** peptidoglycan, divisome, penicillin-binding protein 1b (PBP1b), FtsN, lipid II, bacteria, cell division, cellular regulation, cell surface enzyme, cell wall

## Abstract

Peptidoglycan (PG) is an essential constituent of the bacterial cell wall. During cell division, the machinery responsible for PG synthesis localizes mid-cell, at the septum, under the control of a multiprotein complex called the divisome. In *Escherichia coli*, septal PG synthesis and cell constriction rely on the accumulation of FtsN at the division site. Interestingly, a short sequence of FtsN (Leu^75^–Gln^93^, known as ^E^FtsN) was shown to be essential and sufficient for its functioning *in vivo*, but what exactly this sequence is doing remained unknown. Here, we show that ^E^FtsN binds specifically to the major PG synthase PBP1b and is sufficient to stimulate its biosynthetic glycosyltransferase (GTase) activity. We also report the crystal structure of PBP1b in complex with ^E^FtsN, which demonstrates that ^E^FtsN binds at the junction between the GTase and UB2H domains of PBP1b. Interestingly, mutations to two residues (R141A/R397A) within the ^E^FtsN-binding pocket reduced the activation of PBP1b by FtsN but not by the lipoprotein LpoB. This mutant was unable to rescue the Δ*ponB*-*ponA*^ts^ strain, which lacks PBP1b and has a thermosensitive PBP1a, at nonpermissive temperature and induced a mild cell-chaining phenotype and cell lysis. Altogether, the results show that ^E^FtsN interacts with PBP1b and that this interaction plays a role in the activation of its GTase activity by FtsN, which may contribute to the overall septal PG synthesis and regulation during cell division.

Peptidoglycan (PG) is an essential constituent of the bacterial cell wall and a major antibacterial target; it surrounds the cytoplasmic membrane, determines the cell shape, and protects the cell from rupture under internal osmotic pressure. The PG structure consists of glycan strands made of alternating β-1,4–linked GlcNAc and *N*-acetylmuramic acid residues cross-linked by peptides (1). It is assembled using the lipid II (undecaprenyl–pyrophosphoryl–*N*-acetylmuramic acid–(pentapeptide)–GlcNAc), a cell wall precursor, by the GTase activities of the class A penicillin-binding proteins (aPBPs) and SEDS (shape, elongation, division, and sporulation) proteins and cross-linked by the transpeptidase (TPase) activities of class A and class B PBPs (bPBPs) (2, 3, 4). The GTase and TPase activities are coupled within the same aPBPs and also with their bPBPs TPase partners (*e.g.* PBP1a-PBP2, PBP1b-PBP3) (5). Similarly, the GTase activities of the SEDS proteins are regulated by a cognate bPBP (RodA-PBP2 and FtsW–PBP3) (3, 6, 7, 8). The activities of the PBPs were shown to be regulated within multiprotein complexes, allowing a concerted PG synthesis and sacculus enlargement in line with cell cycle progression (9, 10, 11, 12).

Bacteria generally have at least one bifunctional (GTase/TPase) PBP of class A and one monofunctional (TPase only) PBP of class B (2, 13). *E. coli* contains three aPBPs: PBP1a, 1b, and 1c, and two bPBPs: PBP2 and PBP3, the latter being involved in cell elongation and division, respectively (14). PBP1a and PBP1b are major PG synthases and at least one of them is required for cell viability (15). Although the two PBPs are exchangeable, they likely play specific functions during the cell cycle. PBP1a is mainly involved in cell elongation in partnership with PBP2, whereas PBP1b exhibits a preference for cell division in agreement with its enrichment at midcell during cell constriction (12, 16, 17, 18). PBP1b has a modular structure, composed of an N-terminal tail and a transmembrane anchor followed by the GTase and TPase catalytic domains separated by the regulatory UB2H domain. PBP1b was shown *in vitro* to form a ternary complex with FtsW and PBP3 (8), which constitutes the septal synthase subcomplex of the divisome. This complex interacts with the divisome regulatory proteins FtsBLQ, FtsN and the outer-membrane lipoprotein LpoB (9, 10, 11). FtsN is the last essential protein that localizes at the division site; its accumulation through a self-enhanced positive feedback mechanism triggers cell constriction (19). LpoB binds to the UB2H domain of PBP1b and stimulates both of its catalytic activities (20). FtsN and LpoB bind simultaneously to PBP1b and synergistically stimulate the GTase activity of PBP1b (12). The GTase activity of PBP1b is repressed by FtsBLQ (via FtsL) during divisome assembly and the presence of FtsN and/or LpoB suppresses this inhibition (9). Moreover, FtsBLQ (via FtsQ) was also shown to inhibit the TPase activity of PBP3 but not that of PBP1b (9). On the other hand, CpoB interacts with TolA, and both proteins bind to the PBP1b–LpoB complex, between the UB2H and TPase domains, thus inhibiting the TPase activity of PBP1b but not its GTase activity (12). This regulatory system blocks septal PG (sPG) synthesis catalyzed by PBP1b until the maturation of the divisome is signaled by the accumulation of FtsN, which stimulates PBP1b and counterbalances the inhibitory effect of FtsBLQ, thus contributing to sPG synthesis and cell constriction.

From a structural point of view, FtsN is a bitopic membrane protein composed of a small cytoplasmic domain, a transmembrane α-helix and a large periplasmic domain (21, 22). The cytoplasmic domain of FtsN interacts with the 1C subdomain of the cytoplasmic cell division protein FtsA, which is largely responsible for the recruitment of FtsN to the divisome (23, 24). The periplasmic domain of FtsN is further divided into three subdomains: a membrane-proximal portion containing three potential short α-helices, a glutamine-rich central region, and a PG-binding sporulation-related repeat (SPOR) domain at the C terminus, which binds preferentially to glycan chains devoid of stem peptides (25, 26). The region located around α-helix 2 (Leu^75^–Gln^93^, ^E^FtsN) is essential for the function of FtsN (19, 27).

In this work, we have used complementary techniques including fluorescence anisotropy-binding assays, activity assays, cross-linking, and X-ray crystallography and obtained clear evidence that ^E^FtsN region is sufficient for direct binding to PBP1b and the stimulation of its GTase activity *in vitro.* Furthermore, we determined the crystal structure of PBP1b in complex with ^E^FtsN peptide and identified the binding site of ^E^FtsN between the GTase and UB2H domains of PBP1b.

## Results

### ^E^FtsN is sufficient for the stimulation of the GTase activity of PBP1b

**Figure 1:**
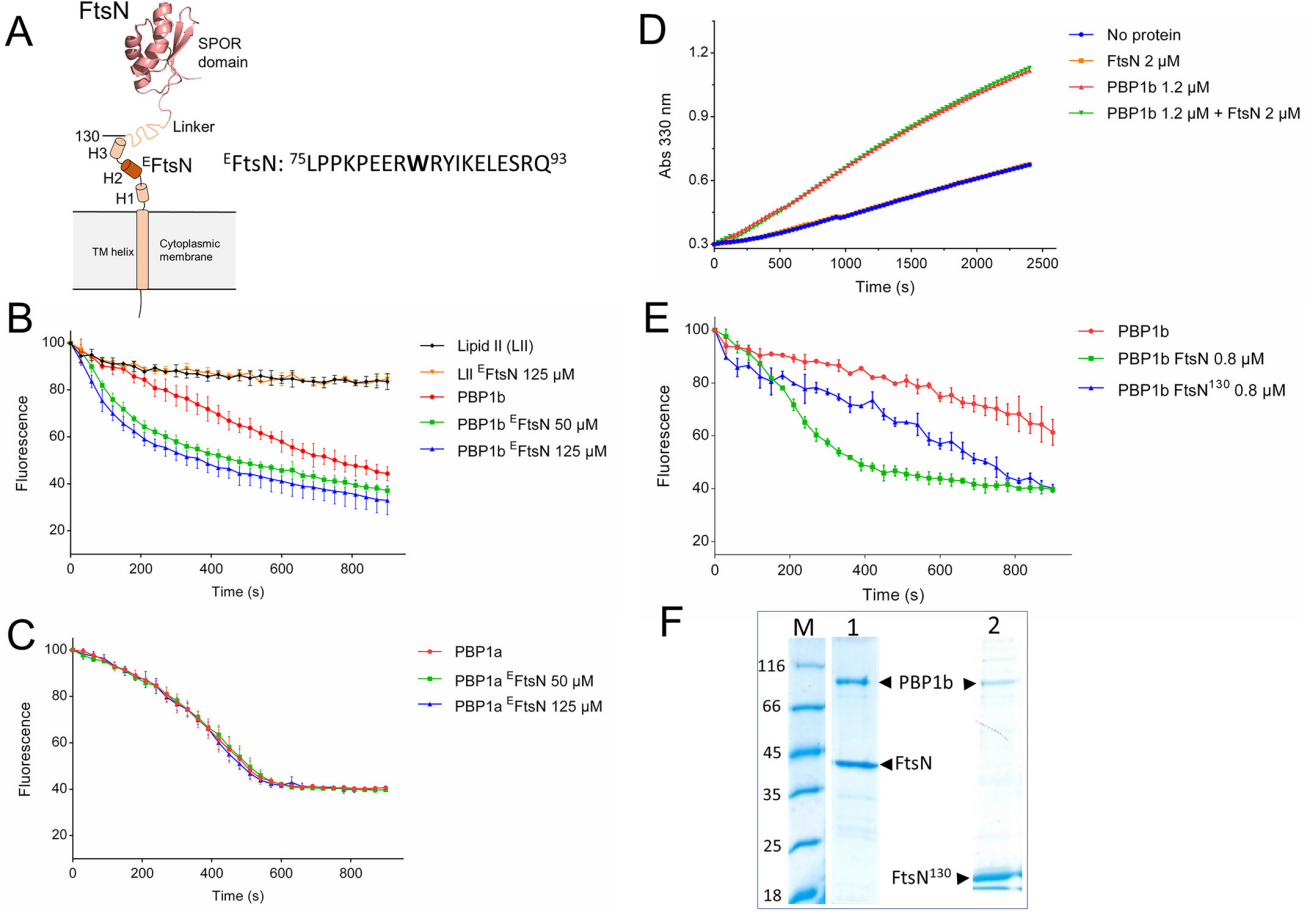
**Activation of PBP1b GTase by ^E^FtsN and FtsN^130^.***A*, schematic representation of FtsN. The protein is composed of a short cytoplasmic tail (positions 1–33) and a transmembrane (TM 34–53) helix followed by three potential short helices (H1 62–67, H2 80–93, and H3 117–123), an unstructured Q-rich linker (positions 129–225) and a C-terminal SPOR domain (positions 243–319) (NMR structure 1UTA (64)). The essential region of FtsN (^E^FtsN) correspond to the sequence Leu^75^–Gln^93^ around H2. The construct FtsN^130^ (Met^1^–Arg^130^) ends just after H3. The *numbers* indicate the amino acid positions of FtsN. *B*, *C*, and *E*, the GTase activity of PBP1b and PBP1a was monitored by continuous fluorescence assay using dansyl-lipid II (LII) as substrate. The *error bars* represent the values as means ± S.D. of three experiments. ^E^FtsN specifically stimulates the GTase activity of PBP1b (*B*) but has no effect on PBP1a (*C*). *D*, the activity of the TPase domain of PBP1b was monitored by following the hydrolysis of S2d in the presence and absence of FtsN (a representative of three independent experiments). FtsN has no effect on the S2d hydrolysis by PBP1b. *Abs 330 nm*, absorbance at 330 nm). *E*, comparison of the stimulation of the GTase activity of PBP1b by FtsN and the truncated form FtsN^130^. *F*, SDS-PAGE analysis of co-expression and co-purification of HisFtsN-PBP1b (*lane 1*) and HisFtsN^130^-PBP1b (*lane 2*). *Lane M*, molecular mass marker.

The stimulation of PBP1b *in vitro* by FtsN is well-established (5, 28). *In vivo*, a small periplasmic domain of FtsN (^E^FtsN: Leu^75^–Gln^93^) (Fig. 1*A*) is required and sufficient for function in cell division (27), but the molecular mechanism of PBP1b activation remains largely unknown. The first indication that ^E^FtsN was involved in PBP1b activation originates from the *in vitro* study of the FtsN mutant W83L. This residue change not only impairs FtsN activity *in vivo* (27) but is less able to stimulate PBP1b activity and to suppress FtsBLQ-mediated inhibition of PBP1b GTase activity *in vitro* (9). In addition, the interaction between FtsN^W83L^ mutant and PBP1b was reduced compared with the WT protein. Based on these observations, we prepared the synthetic peptide (^E^FtsN: Leu^75^–Gln^93^) to test its effect on PBP1b. Interestingly, although a higher concentration than full-length FtsN protein was required (50 μm *versus* ∼1 μm), presumably because of the lack of the transmembrane segment (see below), the peptide was able to stimulate the GTase activity of PBP1b (Fig. 1*B*) but has no effect on that of PBP1a (Fig. 1*C*), confirming that ^E^FtsN directly contributes to PBP1b activation. This result is consistent with *in vivo* data showing that ^E^FtsN (GFP fusion) was functional only when overexpressed (19, 27). When the activity of the TPase domain of PBP1b was analyzed using S2d (a mimic of the d-Ala–d-Ala of the natural substrate) as substrate, the addition of FtsN had no effect (Fig. 1*D*), indicating that the protein only modulates the GTase activity directly but not the TPase activity of PBP1b.

It was previously shown, using different variants of FtsN (28), that multiple interaction sites contribute to the binding with PBP1b (28). The soluble form (sFtsN, Δ1–57), lacking the cytoplasmic tail and the transmembrane (TM) anchor, displayed significantly reduced stimulatory effect on the PBP1b activity (29). To compare the *in vitro* activity of the membrane-bound proximal region containing the three potential α-helices with the full-length protein, we have prepared a truncated variant of FtsN (FtsN^130^ encompassing residues Met^1^ to Arg^130^) (Fig. 1, *A* and *F*). This variant was found to bind PBP1b and to stimulate its GTase activity to a lesser extent compared with full-length protein but was much more efficient in PBP1b activation than the ^E^FtsN peptide (Fig. 1, *E* and *F*). Altogether, these data indicate that the N-terminal region, including the TM segment, is important for high-affinity binding and efficient activation of PBP1b. In addition, the central Gln-rich region and SPOR domain seem to contribute to the optimal activation of PBP1b.

### ^E^FtsN interacts specifically with PBP1b but not with PBP3, FtsW–PBP3, or FtsBLQ

**Figure 2:**
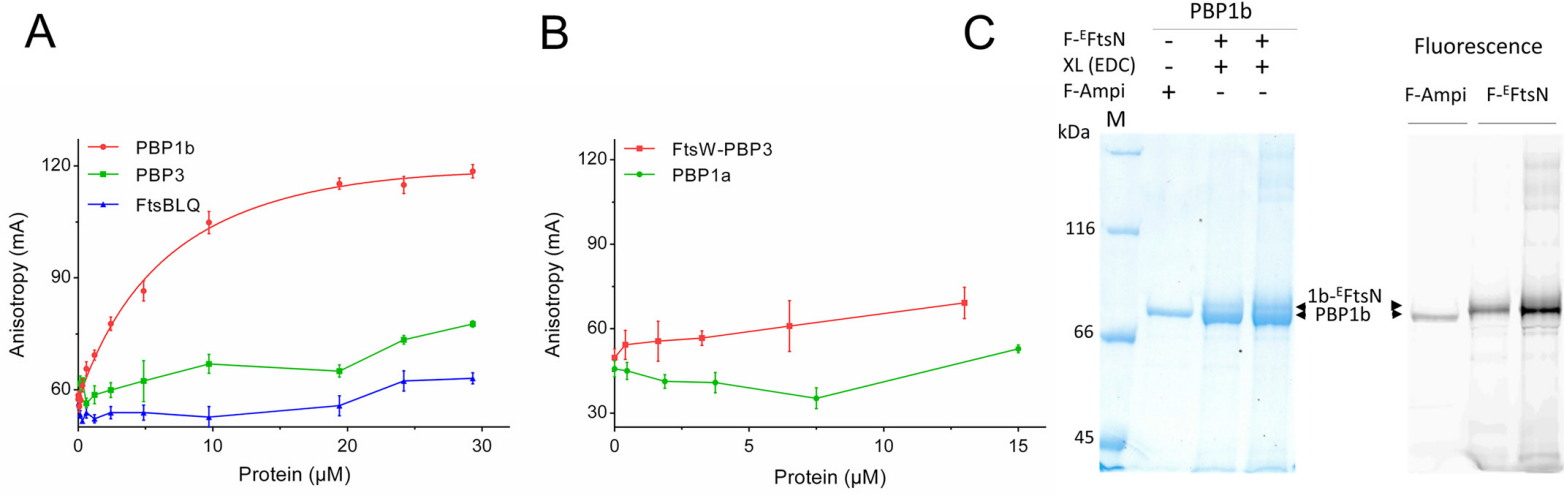
**Binding assays between ^E^FtsN and PBP1b.***A*, direct binding of fluorescent ^E^FtsN (*F-^E^FtsN*) peptide to PBP1b using FA assay. FA (in mA units) is plotted as a function of protein concentrations. *A* and *B*, no significant binding is observed between the probe and PBP3, FtsBLQ complex, FtsW–PBP3 complex, or PBP1a. The *error bars* represent the values as means ± S.D. of three experiments. *C*, SDS-PAGE analysis of cross-linking (XL) adducts between PBP1b (*1b*) and F-^E^FtsN peptide (indicated by the *upper arrows*) using protein:cross-linker molar ratio of 1:1000 (*lane 2*) and 1:2000 (*lane 3*). *Left panel*, SDS-PAGE stained with Coomassie Blue. *Right panel*, fluorescence imaging. F-Ampi depicts PBP1b labeled with fluorescent ampicillin. *Lane M*, molecular mass marker.

To measure the binding between ^E^FtsN and PBP1b or other divisome proteins, a fluorescent peptide (FITC-Lys^69^–Gln^93^) containing a FITC fluorophore attached at the N terminus of the minimal sequence (Leu^75^–Gln^93^) via six additional amino acids of FtsN (to avoid interference with binding) was prepared and used as a probe to develop a fluorescence anisotropy (FA) assay. Interaction studies with PBP1b show a high increase of the FA signal, and fitting of the graph allowed the determination of a dissociation constant *k_d_* of 8.1 ± 1.7 μm (Fig. 2*A*). In contrast, no significant change in the FA signal was observed with PBP1a, PBP3, FtsW–PBP3, and FtsBLQ complexes (Fig. 2, *A* and *B*). This result is consistent with direct and specific interaction of ^E^FtsN with PBP1b and suggests that ^E^FtsN alone is not sufficient for direct interaction with FtsW–PBP3 or FtsBLQ as previously suggested (27). This result is consistent with the absence of strong interaction between FtsN and FtsBLQ and with the fact that FtsN has no effect on the activity of PBP3 (9).

To further characterize the interaction between ^E^FtsN and PBP1b, we performed cross-linking experiments using the FITC- Lys^69^–Gln^93^ peptide to facilitate the visualization of the adduct. The heterobifunctional cross-linking agent 1-ethyl-3-(3-dimethylaminopropyl)-carbodiimide (EDC), a zero-length cross-linker that couples carboxyl groups to primary amines, efficiently coupled PBP1b to the fluorescent peptide (Fig. 2*C*). SDS-PAGE, followed either by Coomassie staining or fluorescence labeling, revealed a band slightly higher (PBP1b + peptide) than PBP1b alone labeled with fluorescent ampicillin. The band intensity increases when the concentration of the cross-linker was increased. This result further confirms that ^E^FtsN interacts with PBP1b.

### Crystal structure of PBP1b in complex with ^E^FtsN

**Figure 3:**
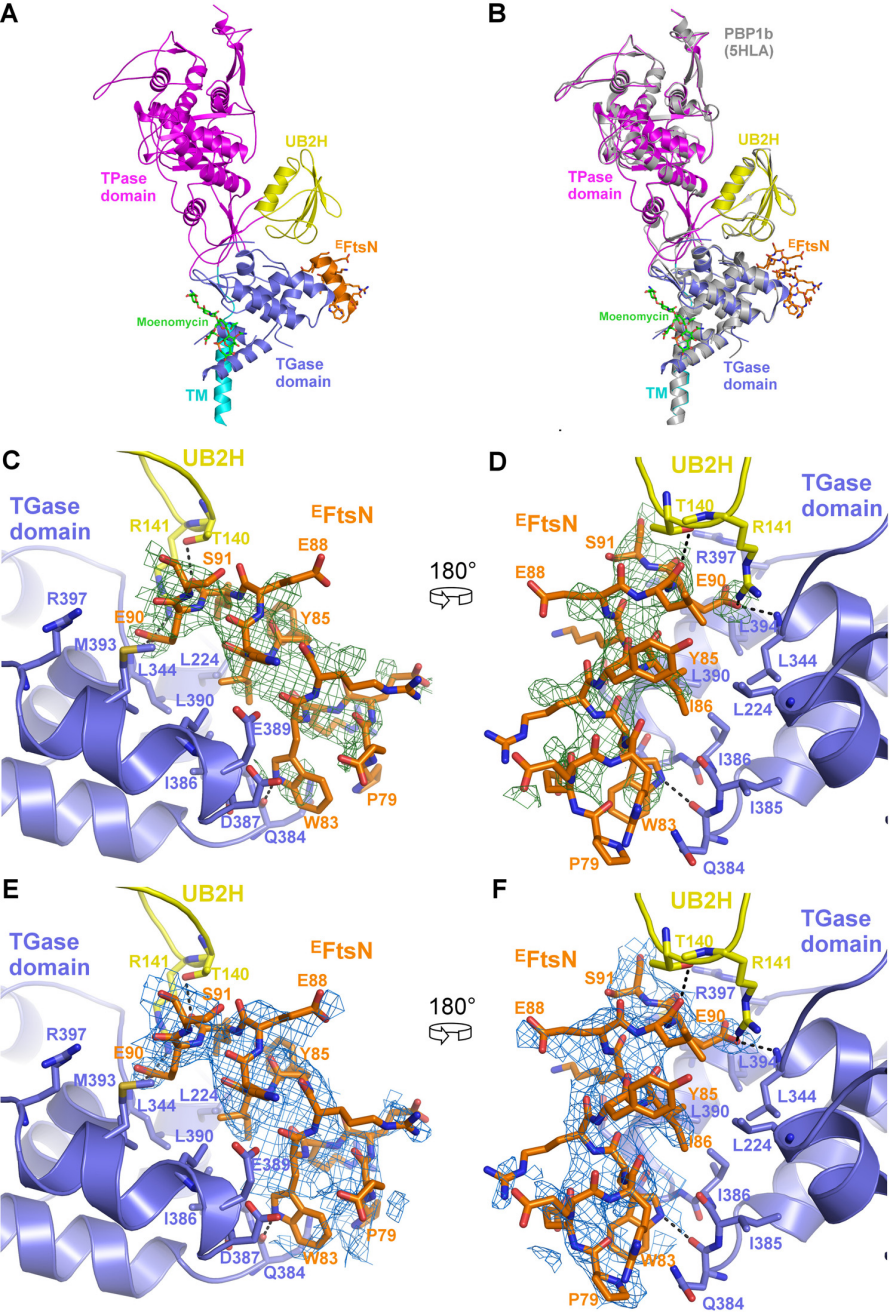
**Crystal structure of PBP1b in complex with ^E^FtsN peptide.***A*, cartoon representation of the PBP1b–^E^FtsN. The TM, UB2H, GTase, and TPase domains are shown in *cyan*, *yellow*, *blue*, and *magenta*, respectively. The ^E^FtsN peptide is shown in *orange*. This structure was obtained in the presence of moenomycin A shown in *green sticks*. *B*, superimposition of the obtained structure (same color code as in *A*) with that of that of published PBP1b structure (PDB code 5HLA) in *gray*. *C* and *D*, details of the interactions between ^E^FtsN represented as *orange sticks* and the GTase (*blue*) and UB2H (*yellow*) domains of PBP1b presented in two orientations at 180° of each other. H-bonds are displayed as *black dashed lines*. The *F*_o_ − *F*_c_ electron-density map obtained before modeling of the peptide is shown around ^E^FtsN at a 2 σ level in *green*. *E* and *F*, same as *C* and *D* with the 2*F*_o_ − *F*_c_ electron density map obtained at the end of the refinement.

Having strong evidence of the binding of ^E^FtsN to PBP1b, we performed co-crystallization assays between PBP1b (positions 58–804), in both the presence and the absence of moenomycin A, which helps in the crystallization of PBP1b, and purified ^E^FtsN (Leu^75^–Gln^93^) peptide to identify the binding site of this domain on PBP1b. Crystals, which were only obtained in the presence of moenomycin A, belong to the space group P2_1_2_1_2 (Table 1), similar to those of previously reported for PBP1b structures (30, 31). The diffraction data were anisotropic, and therefore additional processing was carried out with Staraniso (32). The three axes of the ellipsoid used to cutoff the resolution were 2.3, 4.3, and 2.3 Å. The structure was solved to 2.4 Å resolution by molecular replacement and showed an additional electron density between the GTase and the UB2H domains (Fig. 3, *A–D*). Because of its initial low quality, an attempt to place a helix in both directions and an elongated conformation of the main chain were initially tested. The best solution was selected based on the *R*_free_ and the improvement of the electron density after refinement. The side-chain attribution was done using the secondary structure prediction of ^E^FtsN, with Pro^79^ and Ser^91^, respectively, capping the N- and C-terminal ends of the helix. An attempt at shifting the sequence in both directions on the helix did not lead to improved *R*_free_ or electron density. This 13-amino acid helix mostly interacts with the GTase domain and runs approximately parallel to its last α12 helix. Three hydrogen bonds are observed with PBP1b, between the side chain of Trp^83^ and the main-chain carbonyl of Gln^384^ (α11α12 loop, GTase domain), between the main-chain carbonyl of Leu^89^ and side-chain hydroxyl of Thr^140^ (α1β2 loop, UB2H domain), and between the Glu^90^ side chain and the Arg^141^ side chain (α1β2 loop, UB2H), as well as the Leu^344^ main chain (α9α10 loop, GTase) (Fig. 3, *C–F*). In addition, Tyr^85^, Ile^86^, and Leu^89^ from ^E^FtsN form a hydrophobic cluster with Leu^224^ (α2), Leu^344^ (α9α10 loop), Ile^386^, Leu^390^, and Leu^394^ (α12) from the GTase domain (Fig. 3, *C–F*).

**Table 1:** X-ray crystallographic data collection and refinement statistics of *E. coli* PBP1b in complex with ^E^FtsN

	PBP1b–^E^FtsN
**Data collection**	
Space group	P2_1_2_1_2
*a*, *b*, *c* (Å)	63.1, 283.0, 62.7
α, β, γ (°)	90, 90, 90
Resolution range (Å)^a^	47.2–2.4 (2.51–2.4)
/<σ*I*>^a^	8.1 (1.5)
Completeness elliptical (%)^a^	95.5 (85.9)
Completeness spherical (%)^a^	57.1 (22.3)
Redundancy^a^	8.5 (7.2)
**Refinement**	
Resolution range (Å)	47.2–2.4
No. of unique reflections	25,830
*R*_work_ (%)	22.5
*R*_free_ (%)	25.6
No. atoms	
Protein	5525
Ligands	77
Water	55
RMSDs from ideal stereochemistry	
Bond lengths (Å)	0.01
Bond angles (°)	1.5
Mean B factor (Å^2^)	
Protein	78.2
Ligands	150
Water	36.1
Ramachandran plot (%)	
Favored region	94.2
Allowed regions	5.4
Outlier regions	0.4


a Values in parentheses are related to high resolution shell.

Compared with other PBP1b structures available, no major conformation change was observed upon ^E^FtsN binding with a RMSD of 0.85 Å over 685 common α carbons (PDB code 5HLA) (Fig. 3*B*). The differences are however larger in the GTase domain (RMSD 1.0 Å) than in the TPase (RMSD 0.56 Å) and the UB2H (RMSD 0.38 Å) domains. In the structure of the PBP1b–^E^FtsN complex, the GTase domain is also characterized by overall poorer electron densities compared with the rest of the protein as well as, compared with the GTase domains of other structures available. Of note, the crystals were only obtained in the presence of moenomycin A; therefore it is possible that this ligand may prevent the conformational change induced by the peptide. Nevertheless, we observed a significant destabilization of the GTase domain. This is materialized by a higher mean B factor for α carbons of the GTase domain (125 Å^2^) compared with other PBP1b structures (75 Å^2^ for 5HLA), whereas the TPase and UB2H domains are characterized by lower and more similar values (50 and 48 Å^2^, respectively, *versus* 37 and 46 Å^2^ in the 5HLA structure). This could indicate that the conformational change responsible for the increased GTase activity may indeed be hindered by either the binding of moenomycin A required for crystallization and/or the crystal packing, which strongly traps the GTase domain in an inactive state, whereas the peptide attempts to displace it to an active state.

### Mutations in the ^E^FtsN-binding pocket reduce activation of PBP1b by FtsN and induce cell elongation and lysis

**Figure 4:**
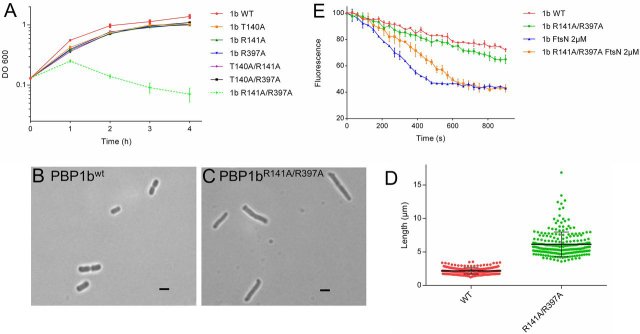
***In vitro* and *in vivo* activities of the PBP1b mutants.***A*, complementation assays of *E. coli* EJ801 strain (Δ*ponB-ponA^ts^*) with the plasmids carrying *ponB* (*1b WT*) gene or *ponB* mutants at nonpermissive temperature (42 °C). *B* and *C*, microscopy analysis of the EJ801 cells expressing PBP1b^WT^ (*B*) or PBP1b^R141A/R397A^ (*C*) after 1 h at 42 °C. *D*, cell length statistics evaluation of EJ801 expressing PBP1b^WT^ (*n* = 250) or PBP1b^R141A/R397A^ (*n* = 250). *E*, comparison of the GTase activation of PBP1b^WT^ (*1b WT*) and PBP1b^R141A/R397A^ by FtsN using continuous fluorescence assay. The *error bars* represent the values as means ± S.D. of three experiments.

To confirm the importance of the ^E^FtsN-binding site in PBP1b activation and the attempt to establish a link between this site and cell division, we addressed the roles of residues Thr^140^ and Arg^141^ from the UB2H domain side of the binding cavity that interact with ^E^FtsN via their side chains and the residue Arg^397^ from the GTase domain side of this cavity, which participate in the same positively charged residues cluster as Arg^141^ and could contribute to the interaction with E90 of ^E^FtsN according to other PBP1b structures. All these residues were modified to Ala, and single or double mutations were introduced in the *pon*B gene. The activity of the mutants was first evaluated by complementation experiments using *E. coli* EJ801 (Δ*ponB*-*ponA*^ts^) (33) as a host strain (results in 10 g/liter LB medium or low salt 0.5 g/liter LB were comparable). The single mutants T140A, R141A, and R397A and the double mutants T140A/R141A and T140A/R397A were able to restore the growth of the strain at the nonpermissive temperature (42 °C) (Fig. 4*A*), and the cells exhibited normal phenotype. In contrast, the double mutant PBP1b^R141A-R397A^ was unable to rescue *E. coli* EJ801 at 42 °C (Fig. 4*A*). This double mutant was stable at 42 °C, and its GTase and Bocillin-binding activities were not affected after 1 h of incubation at this temperature (Fig. S1). The observation of the cells after 1 h at the nonpermissive temperature showed a mild elongated phenotype (L: 6.14 ± 1.84 μm *versus* 2.17 ± 0.46 μm) before lysis (Fig. 4, *B–D*). Moreover, although the purified PBP1b^R141A/R397A^ mutant showed a minor or no difference in the GTase activity compared with the WT protein, its activation by FtsN was reduced by 2-fold (Fig. 4*E*). Interestingly, the activation of PBP1b^R141A/R397A^ by LpoB remained unchanged (Fig. S2). These results indicate that, as a consequence of the mutations in the ^E^FtsN-binding site, the activation of PBP1b by FtsN was reduced, causing defects in sPG integrity and cell division. This strongly confirms the link between PBP1b and FtsN during cell division and demonstrates the role of ^E^FtsN in the activation of its GTase activity.

## Discussion

The mechanisms of *E. coli* septal PG synthesis and regulation are complex and involve several trans-envelope factors, with FtsA, FtsBLQ, FtsW, PBP3, and FtsN playing direct and essential roles in these processes (34, 35, 36). FtsW and PBP3 are the septum-specific GTase and TPase, respectively (37, 38, 39). Notably, PBP1b was shown to localize to the division site and to interact with several core divisome proteins, and its activity, which contributed ∼50–70% of sPG synthesis in the absence of the other GTase (PBP1a, PBP1c, and MtgA), became essential (8, 9, 12, 18, 28, 37, 40). FtsN, on the one hand, and particularly its essential domain ^E^FtsN (27), plays a major role in the initiation of sPG synthesis that governs cell constriction, but what exactly ^E^FtsN does and its ultimate target remained unclear. On the other hand, biochemical data have shown that FtsN interacts with the major PG synthase PBP1b and stimulates its GTase activity (5, 28). Using different techniques including FA-binding assay, activity assay, cross-linking, X-ray crystallography, mutagenesis, and complementation, we provide evidence that ^E^FtsN region is sufficient for direct binding to PBP1b and the partial stimulation of its GTase activity. Importantly, the crystal structure of the PBP1b–^E^FtsN complex allowed the identification of the binding site of ^E^FtsN being located between the GTase and UB2H domains of PBP1b. The GTase domain accounts for most of the interaction with ^E^FtsN, but two residues (Thr^140^ and Arg^141^) in the α1β2 loop of the UB2H domain pointing toward the GTase domain are also involved in the binding. This is consistent with the activation of the GTase but not the TPase activity of PBP1b by FtsN. The binding site of ^E^FtsN is distinct from that of LpoB, which binds the exposed surface of UB2H domain (20, 41). This is supported by the fact that FtsN and LpoB bind simultaneously to PBP1b and that the stimulation of the GTase activity of PBP1b by FtsN was synergistic with the stimulation by LpoB (5).

Furthermore, the structure reveals that the conserved residues Trp^83^, Tyr^85^, and Leu^89^ of ^E^FtsN, which were shown to be important for the *in vivo* function of the protein (27), together with Ile^86^ and Glu^90^ are directly involved in the interaction with PBP1b. Interestingly, the mutant FtsN^W83L^ was shown to exhibit reduced binding and activation of PBP1b (9). The double mutation R141A/R397A in the ^E^FtsN-binding pocket of PBP1b reduces the activation of PBP1b by FtsN but not by LpoB. In addition, this mutant was unable to rescue Δ*ponB*-*ponA*^ts^ strain at nonpermissive temperature and induced a mild cell-chaining phenotype and cell lysis, indicating sPG synthesis and division defects. These data suggest that the observed chaining phenotype is the result of the partial suppression of the ^E^FtsN-binding site. Altogether, the present results reveal that PBP1b, the major class A PBP, is one of the binding targets of ^E^FtsN and that ^E^FtsN is an important determinant for the stimulation of the GTase activity of PBP1b by FtsN. This could contribute to sPG synthesis regulation during cell division. However, our assays could not detect direct interaction between ^E^FtsN and FtsW–PBP3 or FtsBLQ, as previously proposed (27), but this does not exclude that such an interaction occurs with one of these proteins when ^E^FtsN is displayed in the context of the full-length FtsN or an alternative membrane-bound variant.

Overall, our results bridge the gap between the existing biochemical and *in vivo* data (9, 19, 27, 28, 42) and provide additional information to the general model of regulation of cell division in *E. coli*. FtsN seems to play multiple and intricate roles (regulation of sPG synthesis and late hydrolysis) (19, 42) that are coordinated with events on both sides of the cytoplasmic membrane, starting early in the cell cycle via its interaction with FtsA in the cytoplasm (22, 43, 44, 45, 46) and the gradual accumulation throughout the cell cycle (47), using a self-enhanced positive feedback mechanism (19). During divisome assembly, the GTase activity of PBP1b (as well as the TPase activity of PBP3) is kept inactive by the FtsBLQ complex until FtsN reaches a critical threshold, which enables it to relieve this inhibition, probably by disrupting the interaction with FtsBLQ, and to restore PBP1b activity (via direct interaction with ^E^FtsN) (9). This contribute to the global sPG synthesis, probably in coordination with the FtsW–PBP3 complex (3, 8, 37). sPG synthesis then recruits the amidases that hydrolyze sPG and trigger cell constriction (42); this creates a high-affinity binding substrate (denuded PG) of the SPOR domain, allowing the recruitment of more FtsN, and accelerates the constriction process (25, 26).

In *E. coli*, cell wall constriction depends mainly on sPG synthesis and not on FtsZ treadmilling (40, 48). PBP1b was shown to play important role in sPG synthesis complementary to that of FtsW and PBP3 (37, 40) and to exhibit two populations: fast- and slow-moving fractions (49). The slow-moving fraction represents the enzymes catalytically active in PG synthesis. FtsN also exhibits dynamic behavior, and its localization was shown to be spatially separated from constricting FtsZ-ring (50). Because FtsN, a specific divisome protein, binds and activates PBP1b, it may play a role in the transition of PBP1b from the fast and low-activity state to the slow-moving processive enzyme at the division site.

## Experimental procedures

### Bacterial strains, plasmids, and growth conditions

#### Growth conditions

*E. coli* C43 (DE3) or Lemo21 (DE3) transformants were grown in LB or 2× YT medium supplemented with the appropriate antibiotic: ampicillin (100 µg/ml) (from MP Biomedicals), chloramphenicol (30 µg/ml) (from Sigma), or kanamycin (50 µg/ml) (from MP Biomedicals).

#### Reagents

Dansyl-lipid II was prepared as previously described (51, 52). Fluorescein-labeled ampicillin was prepared as previously described (53). FITC-labeled peptide (FITC-Lys^69^–Gln^93^) KVTGNGLPPKPEERWRYIKELESRQ and unlabeled peptide (Leu^75^–Gln^93^) LPPKPEERWRYIKELESRQ were purchased from Synpeptide (Shanghai, China). They were solubilized in 50 mm Tris-HCl, pH 8.0, and stored at −20 °C until use. Moenomycin A was a gift from Aventis (Romainville, Paris, France).

### Plasmid construction

The complete list of the plasmids used in this study is given in Table S1. All point mutations were introduced using the Q5 site-directed mutagenesis kit (New England Biolabs). The primers used in this study are shown in Table S2; they were purchased from Eurogentec (Angleur, Belgium). Details for plasmid constructions are described in the supporting information.

### Expression and purification of proteins

These proteins were purified as previously described: PBP1bγ (54), FtsN and FtsBLQ (9), PBP3 and FtsW–PBP3 (8), PBP1a (55), and LpoB (41). Further details for protein purifications that are not described elsewhere are provided in the supporting information. Of note, all the proteins are stable, PBP3 binds fluorescent ampicillin and hydrolyzes the substrate analog S2d (9), and the FtsW–PBP3 complex was shown to bind lipid II (56).

### FA assay

FA experiments were performed to determine the binding affinity of the ^E^FtsN peptide, consisting of the FITC-Lys^69^–Gln^93^ used as probe, to PBP1bγ. Serial dilutions of the proteins in their specific buffer were prepared in 384-well plates, and the probe was added at 100 nm final concentration in a final volume of 30 μl. The mixtures were incubated for 30 min at 25 °C, and the FA signals were recorded using an Infinite® F Plex (Tecan, Männedorf, Switzerland) equipped with polarization filter with excitation wavelength at 485 nm and emission at 535 nm. FA values were calculated using the equations *FA* = (*I*_‖_ − *G*·*I*_⊥_)/*I*_‖_ + 2*G*·*I*_⊥_), where *I*_‖_ is the fluorescence intensity of emitted light parallel to excitation, *I*_⊥_ is the fluorescence intensity of emitted light perpendicular to excitation, and *G* is the correction factor that correct for instrument bias. The *G* factor is experimentally determined using the probe alone. For *K_d_* determination, the fluorescence anisotropy data were analyzed by nonlinear curve fitting using GraphPad Prism 6.0 software as described (56).

### Continuous fluorescence GTase assay

The GTase activity assays with dansyl-lipid II as substrate were performed in a medium binding black 96-well microplate (Greiner Bio One) as described (57). The samples contained 10 μm dansyl-lipid II, 50 mm HEPES-NaOH, pH 7.5, 200 mm NaCl, 10 mm CaCl_2_, 0.085% of decyl-PEG, 20% of DMSO, and 1 unit of *N*-acetylmuramidase of *Streptomyces globisporus* (Sigma). The ^E^FtsN peptide Leu^75^–Gln^93^ was solubilized in 50 mm Tris-HCl, pH 8.0, and used at 50 and 125 μm concentrations. The proteins FtsN and FtsN^130^ were used at 0.8 μm. The reactions were initiated by the addition of 30 nm PBP1bγ (100 nm for PBP1a) and monitored by following the fluorescence decrease over 20 min at 30 °C using an Infinite 200 PRO microplate reader (Tecan, Männedorf, Switzerland) with excitation wavelength at 340 nm and emission at 520 nm.

### Effect of FtsN on the hydrolysis of S2d by PBP1b

The activity of the TPase domain of PBP1b was measured in the presence of S2d (analog of the peptide moiety) as a mimic of donor substrates as previously described (58, 59). The assay was performed in a UV-Star 96-well microplate (Greiner Bio One) at 37 °C in the presence of 50 mm phosphate buffer, pH 7.0, 2.0 mm S2d, 3.2 mm 4,4′-dithiodipyridine, and 1.2 μm PBP1b. The absorbance at 330 nm was monitored with an Infinite M200 Pro microplate reader (Tecan, Männedorf, Switzerland). FtsN was used at 2 μm to test its effect on the PBP1b activity. The experiments were repeated three times with reproducible results.

### Cross-linking experiments

PBP1bγ (10 μm) was incubated with a 10-fold excess of FITC-^E^FtsN peptide and two conditions of the heterobifunctional cross-linker EDC (protein:EDC molar ratio 1:1000 and 1:2000) in buffer containing 50 mm HEPES-NaOH, pH 7.5, 0.3 mm NaCl, 0.7% CHAPS. The mixture was incubated for 2 h at room temperature and analyzed by SDS-PAGE followed by fluorescence imaging using a Typhoon Trio+ imager and Image Quant TL software (GE Healthcare). The PBP1b control (2 μm) was incubated with fluorescent ampicillin 10 μm for 30 min at 37 °C prior to SDS-PAGE.

### Crystallization, data collection, and structure determination

Crystallization was carried out using the hanging drop vapor diffusion method at 20 °C. The PBP1b (Lys^58^–Ser^804^) concentration was 20 mg/ml in the buffer solution 20 mm Tris-HCl, pH 8.0, 0.3 m NaCl, and 4.5 mm DM and contained 1:1 molar ratio of moenomycin A and a synthetic peptide corresponding to ^E^FtsN (LPPKPEERWRYIKELESRQ) in a 3:1 molar ratio. The crystals were grown in drops made of 2 µl of protein solution and 2 µl of precipitant solution containing 0.1 m (NH4)_2_SO_4_, 0.3 m sodium formate, 0.1 m Tris-HCl, pH 7.8, 3% (w/v) low-molecular-weight poly-γ-glutamic acids, and 20% (v/v) PEG 550 monomethyl ether. The cryoprotectant solution was made of 22% (w/v) PEG 6000 and 30% (v/v) PEG 400. The diffraction data were collected on the Proxima 1 beamline of the Soleil Synchrotron (Paris-Saclay). The data were indexed, integrated, and scaled using XDS and reached a 2.4 Å resolution (60). Because of the anisotropy of the data, additional processing was carried out with Staraniso (32). The three axes of the ellipsoid used to cutoff the resolution were 2.3, 4.3, and 2.3 Å. The crystal used belongs to the orthorhombic P2_1_2_1_2 space group. The structure was solved by molecular replacement with Phaser (61) using the PBP1b structure of PBD code 3VMA as search model (30). The refinement and model building cycles were respectively performed with buster (BUSTER version 2.10.2, Global Phasing Ltd., Cambridge, UK) and Coot (62). A summary of the relevant statistics of the data collection and refinement is given in Table 1. The figures were prepared using PyMOL (PyMOL molecular graphics system, version 1.7.4.3 Enhanced for Mac OS X, Schrödinger, LLC). The coordinates and structure factors of the structure have been deposited in the Protein Data Bank under code 6YN0.

### Complementation assay

To test the activity of the PBP1b mutants *in vivo*, we tested their ability to complement *E. coli* strain EJ801, which lacks PBP1b and has a thermosensitive PBP1a (33). EJ801 cells were transformed with the corresponding plasmids and grown in LB at 30 °C with 50 µg/ml kanamycin. The preculture was then diluted in 30 ml of fresh LB (10 g/ml NaCl) or LB with 0.5 g/ml NaCl medium to an *A*_600nm_ value of 0.04 and grown at 30 °C until *A*_600nm_ = 0.1–0.3, and the absorbance of the culture was monitored for 4 h at 42 °C.

### Microscopy and image analysis

Samples were taken from the complementation cultures (42 °C) at different time (1-h intervals), and the cells were fixed as described (63). Photographs were taken with a cooled AxioCam MRm (Zeiss) mounted on a Zeiss Axio Imager. Z1 microscope, and the images were acquired in phase-contrast mode using the AxioVision Rel. 4.5 (Zeiss) software. The cell morphologies analysis were determined using ImageJ software (RRID:SCR_003070) running under plugin ObjectJ (RRID:SCR_019190).

## Data availability

All data relevant to this work are contained within this article and the associated supporting information. The structural data are available in RCSB Protein Data Bank under the accession number: 6YN0.
