# Supplementary material for: The bacterial cell division protein fragment EFtsN binds to and activates the major peptidoglycan synthase PBP1b
Source: J Biol Chem. 2021 Jan 13;295(52):18256–65. doi: 10.1074/jbc.RA120.015951 (PMC7939390; doi:10.1074/jbc.RA120.015951)
Supplement: Supplementary file 1 [file mmc1.pdf]

Supporting information

The bacterial cell division protein fragment ^{E}FtsN binds to and activates the major peptidoglycan synthase PBP1b

Adrien Boes[1], Frederic Kerff[1], Raphael Herman[1], Thierry Touze[2], Eefjan Breukink[3], Mohammed Terrak[1]

[1] InBioS-Centre d'Ingénierie des Protéines, Liège University, B6a, Quartier Agora, allée du six Août 11, 4000 Liège 1, Belgium
[2] Université Paris-Saclay, CEA, CNRS, Institute for Integrative Biology of the Cell (I2BC), Gif-sur-Yvette, France.
[3] Membrane Biochemistry and Biophysics, Department of Chemistry, Faculty of Science, Utrecht University, The Netherlands.

***Corresponding author :** Mohammed Terrak,
E-mail: mterrak@uliege.be

Running title: Régulation of PBP1b by FtsN

Keywords: Peptidoglycan, divisome, Penicillin Binding-Protein 1b (PBP1b), FtsN, Lipid II

Plasmids construction

pET28a-PBP1b(K58-S804). The *E. coli pon*B gene encoding the truncated form of PBP1b corresponding to amino acids K58–S804 was amplified by PCR from pDML924 plasmid using the primers 1B_K58 and 1B_S804 (Table S2). PCR product was digested by NdeI/XhoI and ligated into the plasmid pET-28a between the corresponding sites. The resulting plasmid pET28a-PBP1b(K58-S804) encodes for PBP1b (K58-S804) polypeptide with an N-terminal His-tag followed by a thrombin cleavage site.

pET22b-*fts*N^{130}His. The *fts*N1-130 fragment was amplified by PCR from pDML2032 (Table S1) using the primers N_M1/pET22b and N_R130/pET22b (Table S2), digested by NdeI/XhoI and cloned into the plasmid pET22b between the corresponding sites. The encoded protein contains a His-tag at the C-terminus.

pDuet-His*fts*N^{130}-*pon*B. *fts*N1-130 fragment was amplified by PCR from pDML2032 using N_M1/pDuet and N_R130/pDuet (Table S2) and inserted in the MCS1 of the pETDuet1-HisFtsN-PBP1b plasmid between BamHI/ HindIII to replace the full-length *fts*N gene (Table S1). FtsN^{130} contains a His-tag at the N-terminus.

Expression and purification of proteins

PBP1b(58-S804). C43(DE3) cells transformed with pET28-PBP1b(K58-S804) were grown in Luria-Bertani (LB) with kanamycin (50 µg/ml) at 37°C to an A_{600nm} of 0.8. Then, the expression was induced for 3.5 hours by addition of 0.5 mM isopropyl β-D-1-thiogalactopyranoside (IPTG). Cells were collected by centrifugation at 4000 × *g* for 20 minutes at 15°C and resuspended in a buffer containing 20 mM Tris-HCl pH 8.0, 300 mM NaCl and EDTA-free protease inhibitor Cocktail (Roche). The bacterial cells were lysed by three passages through a cell homogeneizer (Emulsiflex C3 Avestin). After centrifugation at 4000 × *g* for 20 minutes at 4°C, the supernatant was recovered and was spun down at 150,000 × g for 1 hour at 4°C and then the membranes were solubilized in 20 mM Tris-HCl pH 8.0, 500 mM NaCl, 10% v/v glycerol, 40 mM *n*-dodecyl-β-D-maltopyranoside (DDM; Inalco) and complete EDTA-free protease inhibitors. The mixture was incubated for 1 hour at room temperature under gentle agitation followed by centrifugation at 150,000 × g for 1 hour at 4°C. The supernatant containing the solubilized membrane proteins was loaded onto a HisTrap column (GE HealthCare) conditioned in buffer A (20 mM Tris-HCl pH 8.0, 300 mM NaCl, 1 mM DDM). After a wash with buffer A supplemented with 100 mM imidazole, the protein was eluted using a linear gradient of imidazole from 100–500 mM. The pure fractions were pooled and desalted on a G25 Sephadex column (GE HealthCare). The N-terminal His-tag was cleaved by the addition of 5 unit of bovine α-thrombin (Sigma**)** per mg of PBP1b during overnight incubation at room temperature. The sample was loaded again onto a HisTrap column conditioned in buffer A, the protein without His- tag was recovered in the flow through and during washing step with buffer A. The protein was concentrated using a 50-kDa cutoff Amicon centrifugation unit. PBP1b was further purified on a Superdex 200 Increase 10/300 GL (GE Healthcare) conditioned with 20 mM Tris-HCl pH 8.0, 300 mM NaCl, 4.5 mM *n*-decyl-β-D-maltopyranoside (DM; Anatrace). The protein was finally concentrated until 20 mg/ml and used for crystallization.

FtsN^{130} and PBP1b-FtsN^{130} complex. C43(DE3) cells transformed with pET22b-*fts*N^{130}His or pDuet-His*fts*N^{130}-*pon*B were grown in Luria-Bertani (LB) with ampicillin (100 µg/ml) at 37°C to an A_{600nm} of 0.8. Then expression was induced for 3.5 hours by addition of 0.5 mM IPTG. Cells were collected by centrifugation at 4000 × *g* for 20 minutes at 15°C and resuspended in a buffer containing 20 mM Tris-HCl pH 8.0, 300 mM NaCl and EDTA-free protease inhibitor Cocktail (Roche). The cells were lysed by three passages through a cell homogeneizer (Emulsiflex C3 Avestin®). After centrifugation at 4000 × *g* for 20 minutes at 4°C, the supernatant was recovered and was spun down at 150,000 × g for 1 hour at 4°C and then the membranes were solubilized in 25 mM Tris-HCl pH 8.0, 500 mM NaCl, 10% v/v glycerol, 40 mM

DDM (Inalco) and complete EDTA-free protease inhibitors. The mixture was incubated for 1 hour at room temperature under gentle agitation followed by centrifugation at 150,000 × g for 1 hour at 4°C. The supernatant containing the solubilized membrane proteins was loaded onto a HisTrap column (GE HealthCare) conditioned in buffer B (25 mM Tris-HCl pH 7.5, 500 mM NaCl, 4 mM DDM). After a wash with buffer B supplemented with 100 mM imidazole, the proteins were eluted using a linear gradient of imidazole from 100–500 mM. The pure fractions were pooled and desalted on a G25 Sephadex column (GE HealthCare).

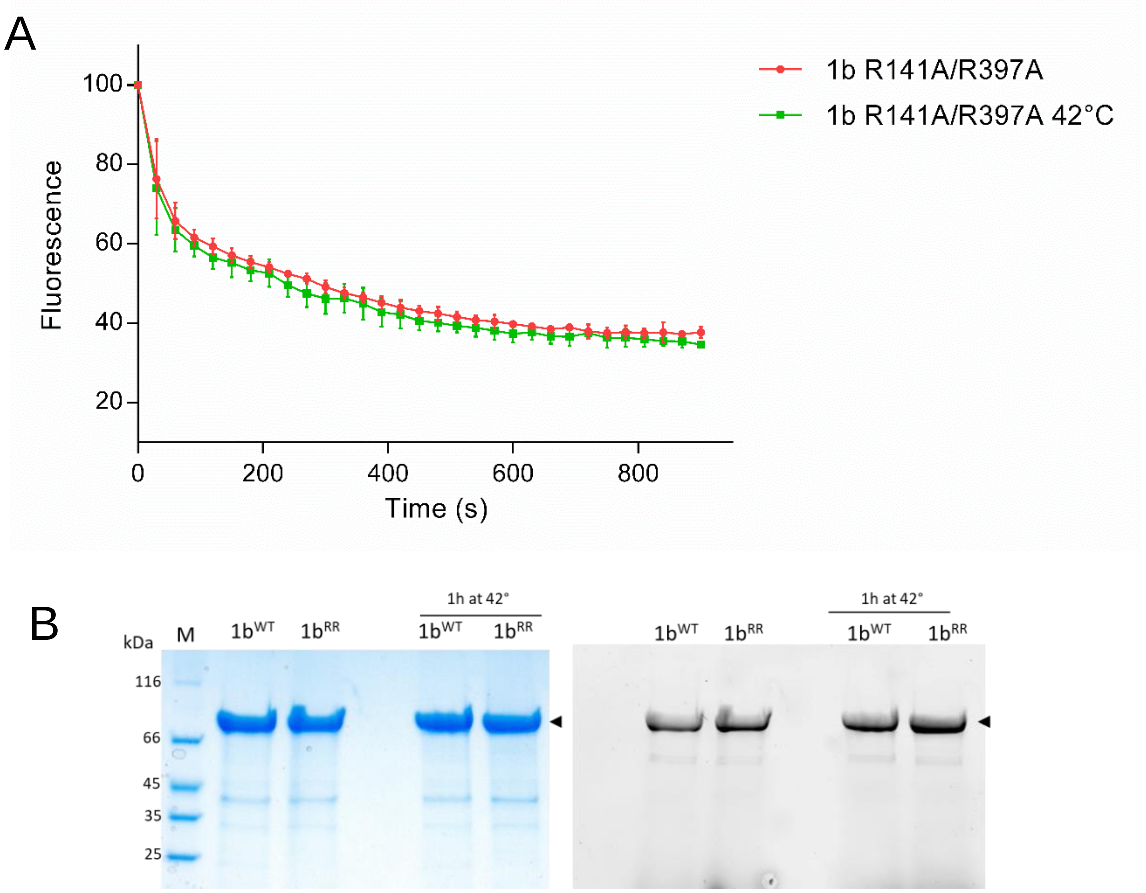

Figure S1. Stability of the double mutant PBP1b$^{R141A-R397A}$ at 42°C. (A) The purified protein was incubated at 30°C or 42°C for 1 h and its GTase activity was monitored at the same temperature by continuous fluorescence assay using dansyl-lipid II (LII) as substrate. The error bars represent the values as mean ± s.d. of three experiments. (B) after 1h at 30°C or 42°C the protein (7 µg) was labelled with Bocillin FL (50 µM) for 30 min at the same temperature and analyzed by SDS-PAGE followed by fluorescence imaging (right) and Coomassie blue staining (left). 1b^{WT} and 1b^{RR} depict PBP1b wild-type and PBP1b double mutant R141A-R397A, respectively. M, Molecular mass marker.

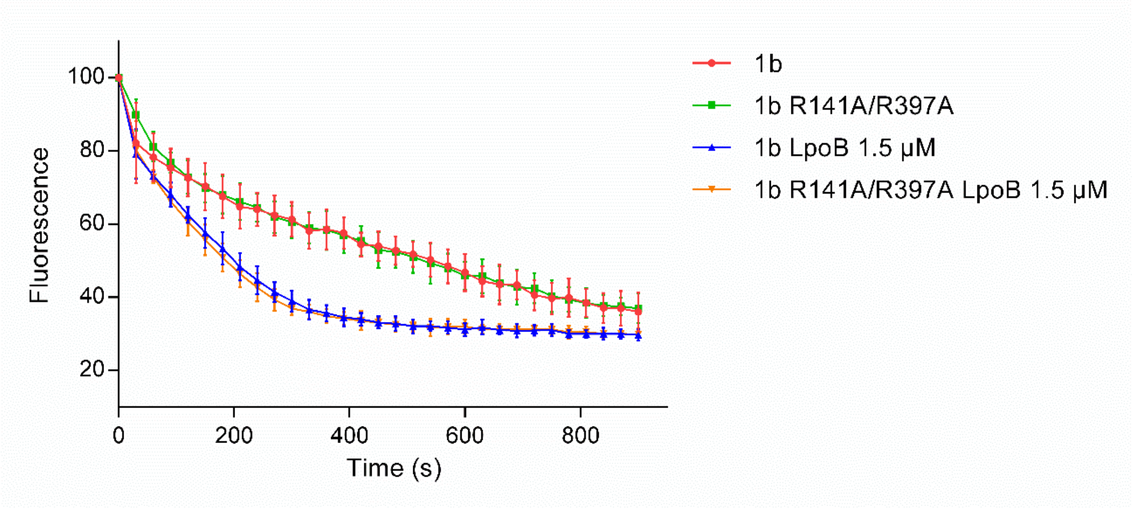

Figure S2. Comparison of the activation of $PBP1b^{WT}$ (1b) and $PBP1b^{R141A\text{-}R397A}$ by LpoB**.** The GTase activity of the $PBP1b^{WT}$ and $PBP1b^{R141A\text{-}R397A}$ was monitored in the presence or the absence of LpoB by continuous fluorescence assay using dansyl-lipid II (LII) as substrate. The error bars represent the values as mean ± s.d. of three experiments.

Table S1. Plasmids used in this study

Plasmid	Description	Reference
pDML924	His-PBP1bγ	(1)
pDML924 (T140A)	His-PBP1bγ (T140A)	This work
pDML924 (R141A)	His-PBP1bγ (R141A)	This work
pDML924 (R397A)	His-PBP1bγ (R397A)	This work
pDML924 (T140A/R141A)	His-PBP1bγ (T140A/R141A)	This work
pDML924 (T140A/R397A)	His-PBP1bγ (T140A/R397A)	This work
pDML924 (R141A/R397A)	His-PBP1bγ (R141A/R397A)	This work
pET28-*pon*B(K58-S804)	His-PBP1b(K58-S804)	This work
pDML2032	FtsN-His	(2)
pET22b-*fts*N130His	FtsN130-His	This work
pTK1A	His-PBP1A	(3)
pRSF-His*fts*BL*Q	His-FtsB/FtsL*/FtsQ	(4)
pDML2494	His-PBP3	(5)
pDuet-His*fts*N-*pon*B	His-FtsN / PBP1b	(4)
pDuet-His*fts*N130-*pon*B	His-FtsN130 / PBP1b	This work

Table S2: Oligonucleotides used in this study

Oligonucleotide name	Sequence 5'→ 3'
1B_K58	GATACGCATCTCGAGTTATGACGGCTGCTGCTGCATCTC
1B_S804	GATACGCATCTCGAGTTATGACGGCTGCTGCTGCATCTC
N_M1/pET22	TATACATATGGCACAACGAGATTATG
N_R130/pET22	TATACTCGAGGCGCATATCAGCCTGC
N_M1/pDuet	TATAGGATCCTGCACAACGAGATTATGTACG
N_R130/pDuet	TATAAAGCTTTTAGCGCATATCAGCCTGC
1B_T140A_F	GTCGAAAATGGCGCGTCCTGGCG
1B_T140A_R	ACCTGACGATACTGGGTC
1B_R141A_F	GAAAATGACCGCGCCTGGCGAATTTACC
1B_R141A_R	GACACCTGACGATACTGG
1B_ R397A_F	GTTGAGTGCCGCGCCGCTGGGGG
1B_ R397A_R	ATGTCATAGAGTTCTTGATCAATAATC
1B_T140A/R141A_F	GTCGAAAATGGCGGCGCCTGGCGAATTTACC
1B_T140A/R141A_R	ACCTGACGATACTGGGTC

Supplementary references

1. Terrak, M., Ghosh, T. K., Van Heijenoort, J., Van Beeumen, J., Lampilas, M., Aszodi, J., Ayala, J. A., Ghuysen, J.-M., and Nguyen-Distèche, M. (1999) The catalytic, glycosyl transferase and acyl transferase modules of the cell wall peptidoglycan-polymerizing penicillin-binding protein 1b of Escherichia coli. *Mol. Microbiol.* 10.1046/j.1365-2958.1999.01612.x

2. Müller, P., Ewers, C., Bertsche, U., Anstett, M., Kallis, T., Breukink, E., Fraipont, C., Terrak, M., Nguyen-Distèche, M., and Vollmer, W. (2007) The Essential Cell Division Protein FtsN Interacts with the Murein (Peptidoglycan) Synthase PBP1B in Escherichia coli. *J. Biol. Chem.* **282**, 36394–36402

3. Born, P., Breukink, E., and Vollmer, W. (2006) In vitro synthesis of cross-linked murein and its attachment to sacculi by PBP1A from Escherichia coli. *J Biol Chem*. **281**, 26985–26993

4. den Blaauwen, T., and Luirink, J. (2019) Checks and Balances in Bacterial Cell Division. *MBio*. 10.1128/mBio.00149-19

5. Piette, A., Fraipont, C., Den Blaauwen, T., Aarsman, M. E. G., Pastoret, S., and Nguyen-Distèche, M. (2004) Structural determinants required to target penicillin-binding protein 3 to the septum of Escherichia coli. *J. Bacteriol.* **186**, 6110–7
